# Responses of gut microbiota in crocodile lizards (*Shinisaurus crocodilurus*) to changes in temperature

**DOI:** 10.3389/fmicb.2023.1263917

**Published:** 2023-11-15

**Authors:** Zhengzhong Lin, Mingxian He, Chunying Zhong, Yuhui Li, Sanqi Tang, Xindan Kang, Zhengjun Wu

**Affiliations:** ^1^Key Laboratory of Ecology of Rare and Endangered Species and Environmental Protection, Guangxi Normal University, Ministry of Education, Guilin, China; ^2^Guangxi Key Laboratory of Rare and Endangered Animal Ecology, Guangxi Normal University, Guilin, China; ^3^College of Food and Biochemical Engineering, Guangxi Science and Technology Normal University, Guangxi, China; ^4^College of Vocational and Technical Education, Guangxi Science and Technology Normal University, Guangxi, China

**Keywords:** *Shinisaurus crocodilurus*, temperature, gut microbiota, responses, 16S rRNA high-throughput sequencing

## Abstract

The gut microbiota plays an essential role in maintaining the health and fitness of the host organism. As a critical environmental variable, temperature exerts significant effects on animal survival and reproduction. Elevated temperatures can influence the composition and function of the animal gut microbiota, which may have potentially detrimental effects on the host. The crocodile lizard (*Shinisaurus crocodilurus*) is an ancient and currently endangered reptile species due to human hunting and habitat destruction. Given the predicted shifts in global temperatures in the next century, it is important to understand how warming affects the gut microbiota of these vulnerable lizards, which remains unclear. To determine how the microbial communities change in crocodile lizards in response to warming, we analyzed the gut microbiota under five temperature conditions (22°C, 24°C, 26°C, 28°C, and 30°C) using 16S rRNA high-throughput sequencing. Results showed that the dominant phyla, Proteobacteria and Bacteroidetes, in gut microbiota were not significantly affected by temperature variations, but increasing temperature altered the structure and increased the community richness of the gut microbiota. In addition, warming changed the abundance of *Pseudomonas aeruginosa* and Actinobacteria, which may have negative effects on the physiological health of the crocodile lizards. Functional prediction analysis demonstrated that the functional pathways enriched in crocodile lizards were mainly related to metabolism, with no significant differences observed in these pathways at KEGG pathway level 1 after warming. These results provide valuable insights into the ecological adaptations and regulatory mechanisms employed by crocodile lizards in response to warming, which may be of benefit for their conservation.

## Introduction

The gut microbiota exhibits remarkable diversity and unique functional characteristics, playing an important role in host development (Sommer and Baeckhed, 2013), metabolism (Flint et al., 2012; Tremaroli and Backhed, 2012), immunity (Kau et al., 2011; Thaiss et al., 2016), and social behavior (Raulo et al., 2018). Maintaining a healthy gut microbiota is vital for the normal physiological functions and overall well-being of the host, with alterations in its composition disrupting normal physiological processes, even contributing to disease (Holmes et al., 2011; Clemente et al., 2012). Despite relatively extensive research on gut microbiota in mammals (Ley et al., 2008a,b), there remains a dearth of studies on reptilian gut microbiota (Colston and Jackson, 2016; Siddiqui et al., 2022), although factors such as captivity (Kohl et al., 2017; Zhou et al., 2020), diet (Jiang et al., 2017), altitude (Zhang et al., 2018), and temperature (Bestion et al., 2017; Sepulveda and Moeller, 2020; Zhang Z. et al., 2022) have been implicated as potential influences.

Long-term monitoring has indicated that the global climate is undergoing considerable changes due to the increase in greenhouse gas concentrations (Hughes, 2000; Root et al., 2003). Global average surface temperatures have risen at a rate of 0.2 ± 0.1°C per decade and are projected to increase by 1.5°C compared to pre-industrial levels by the middle of the 21st century (Hoegh-Guldberg et al., 2019). Global warming has driven the extinction of several amphibian species, with one in six species on Earth now at risk of extinction (Pounds et al., 2006; Urban, 2015). As a critical environmental factor, temperature significantly influences animal growth, development, and reproduction (Gillooly et al., 2002; Angilletta et al., 2010), with global warming posing a particular threat, especially to ambient-temperature reliant reptiles (Root et al., 2003; Xu et al., 2017; Leung et al., 2020). Notably, in reptiles, temperature can exert substantial effects on various ecological and reproductive factors (Meiri et al., 2013), including digestive performance (Pafilis et al., 2007), offspring sex determination (Janzen, 1994; Warner and Shine, 2008), metabolism (Milsom et al., 2008), and athletic ability (Liu et al., 2022). Recent research has also revealed that temperature changes can impact the composition and function of the gut microbiota (Sepulveda and Moeller, 2020; Liu et al., 2022). For example, temperature increases have been shown to reshape the gut microbiota in *Eremias argus*, leading to altered and destabilized composition in response to adaptive states (Zhang Z. et al., 2022). Similarly, an increase of 2–3°C has been found to cause a significant decrease in the gut microbial diversity of *Zootoca vivipara* (Bestion et al., 2017). In addition, an increase of 10°C has been demonstrated to significantly affect the composition and dynamics of the gut microbiota in *Sceloporus occidentalis*, with potential implications for the physiological performance and fitness of natural populations (Moeller et al., 2020). Thus, these studies emphasize the crucial role of temperature in influencing the equilibrium of animal gut microbiota, further underscoring the potential adverse effects of warming on host survival and fitness.

The crocodile lizard (*Shinisaurus crocodilurus*) is an ancient reptile in the family Shinisauridae and order Squamata. It is a class I protected animal in China and is listed as an endangered species on the IUCN Red List of Threatened Species (Yu et al., 2009). This species is a semi-aquatic lizard, preferring mountainous streams within evergreen broadleaf forests and bamboo forests (Li et al., 2019; Yang et al., 2020). The crocodile lizard is distributed in a few isolated sites in southern China (Guangdong Province and Guangxi Zhuang Autonomous Region) and northern Vietnam (Quang Ninh and Bac Giang provinces) (van Schingen et al., 2014, 2016). Due to the pressure of being hunted, environmental changes, and habitat destruction, wild populations of crocodile lizards have declined dramatically, decreasing from approximately 6,000 in 1978 to 1,200 in 2004 across eight wild populations in southern China (Huang et al., 2008, 2014; Jiang et al., 2017). Recent field surveys have also shown that wild populations in Vietnam have decreased to fewer than 150 individuals (van Schingen et al., 2016).

According to previous studies, crocodile lizards prefer relatively low temperatures and they are able to thermoregulate better in the lower thermal quality environment (Yang et al., 2020). In contrast, as a result of increasing temperatures, the offspring survival of crocodile lizards may be hampered (Li et al., 2019). What’s worse, in a future global warming scenario, the potential range of crocodile lizards will continue to shrink (Zhang X. et al., 2022). Therefore, rising ambient temperatures may impose a threat on the survival and reproduction of crocodile lizards. Furthermore, considering the significant impact of the gut microbiota on the survival and fitness of host organisms, it is crucial to examine the factors influencing the gut microbiota of endangered crocodile lizards for their effective conservation. Existing research has revealed the influence of diet and captivity on the community structure of the gut microbiota in crocodile lizards (Jiang et al., 2017; Tang et al., 2020). However, the effects of temperature on the gut microbiota of crocodile lizards remain unexplored. Therefore, we conducted a laboratory experiment to examine the changes in the gut microbiota of crocodile lizards under five temperature gradients (22°C, 24°C, 26°C, 28°C, and 30°C) using 16S rRNA high-throughput sequencing. This study should provide insights into how the gut microbiota of crocodile lizards responds to warming, thereby facilitating effective conservation measures for this endangered species.

## Materials and methods

### Experimental design

The crocodile lizards used in this study were from the Daguishan Crocodile Lizard National Nature Reserve. The experiment was conducted between June and July 2020 in a controlled artificial greenhouse located within the reserve, and the average temperature of outdoor enclosures was 26.06°C ± 1.81°C in June and 26.91°C ± 1.59°C in July. A total of seven healthy adult crocodile lizards of similar age and size were selected from the crocodile lizards breeding pond. The morphological characteristics of the crocodile lizards, including weight, total length, head length, head width, and head height, were measured and recorded (Supplementary Table S1). Additionally, food intake and body temperature of the crocodile lizards was recorded in each temperature experiment (Supplementary Tables S2, S3).

The experimental temperature range was set at 22–30°C, considering their potential reduction in activity or hibernation in lower temperatures and potential adverse effects on their well-being at excessively high temperatures. The experiment comprised a total of five temperature groups (T1: 22°C, T2: 24°C, T3: 26°C, T4: 28°C, and T5: 30°C), with a temperature gradient of 2°C to ensure the accuracy of the temperature control instrument. The experiment was carried out in a controlled artificial greenhouse, where the temperature was adjusted to the designated level using air conditioning and temperature-controlled wooden breeding boxes (50 cm × 30 cm × 30 cm). To simulate the natural breeding pond environment and minimize external interference, an imitation porcelain basin (33 cm × 15 cm × 2 cm) filled with water was placed inside the breeding boxes. Throughout the experiment, all crocodile lizards were provided with consistent food and water under a 12 h/12 h light/dark cycle (on at 07:00, off at 19:00). Sampling was performed on day 8, marking the completion of the temperature gradient experiment. Subsequently, the process was repeated by adjusting the temperature of the breeding boxes to the next designated experimental temperature until all planned sampling procedures were completed.

### Sample collection

Cloacal swabs (Puritan Calgiswab Sterile Urogenital Calcium Alginate Sampler 25-801A50, Puritan Medical Products Company LLC, Guilford, ME, United States) were used for sampling measuring 14 cm, which have been established as a reliable method for non-destructive sampling of gut microbiota in reptiles (Jiang et al., 2017). Following the capture of crocodile lizards, the external cloaca was carefully cleansed with alcohol to reduce the interference of environmental microbiota and ensure that the collected cloacal swabs predominantly contained the gut microbiota. Subsequently, the swab was gently inserted into the cloaca, taking care to insert into the large intestine instead of beyond the coprodeum, rotated 3–5 times to facilitate adherence of gut secretions to the swab, and then withdrawn and placed into a sterile cryopreservation tube. After recording sample details, the sterile cryopreservation tubes were temporarily stored in a −20°C refrigerator, then placed in a −80°C refrigerator upon return to the laboratory.

### DNA extraction, polymerase chain reaction amplification, and sequencing

DNA extraction and sequencing were conducted by the Majorbio Corporation (Shanghai, China), according to established protocols. Total DNA of the gut microbiota was extracted using an E.Z.N.A.^®^ Soil DNA kit (Omega Bio-tek, Norcross, GA, United States). We pulverized the samples and added buffer, and then used magnetic beads to extract DNA. Extract quality was assessed using 1% agarose gel electrophoresis, while DNA concentration and purity were determined using a NanoDrop 2000 UV–vis spectrophotometer (Thermo Fisher Scientific, Wilmington, DE, United States).

Amplification of the hypervariable V3–V4 region of the bacterial 16S rRNA gene was carried out using primer pairs 338F (5’-ACTCCTACGGGAGGCAGCAGCAG-3′) and 806R (5’-GGACTACHVGGGTWTCTAAT-3′) with an ABI GeneAmp 9,700 thermocycler (ABI, CA, United States) (Mori et al., 2014). The PCR assay was as follows: initial denaturation at 95°C for 3 min, 27 cycles of denaturation at 95°C for 30 s, annealing at 55°C for 30 s, and extension at 72°C for 45 s, final extension at 72°C for 10 min, followed by storage at 4°C. For the PCR test, TransGen AP221-02: TransStart FastPfu DNA polymerase was used in a 20-μL reaction system containing 4 μL of 5 × TransStart FastPfu buffer, 2 μL of 2.5 mM deoxy-ribonucleoside triphosphates (dNTPs), 0.8 μL of forward primer (5 μM), 0.8 μL of reverse primer (5 μM), 0.4 μL of TransStart FastPfu polymerase, 10 ng of template DNA, and 20 μL of ddH2O. The PCR assay was performed in triplicate. The PCR products were extracted with 2% agarose gel and purified using an AxyPrep DNA Gel Extraction Kit (Axygen Biosciences, Union City, CA, United States), then subsequently detected using 2% agarose gel electrophoresis, and quantified using a Quantus™ Fluorometer (Promega, United States). The sequencing libraries were then established using a NEXTFLEX Rapid DNA-Seq Kit (Bioo Scientific, Austin, TX, United States). High-throughput sequencing was performed on the Illumina MiSeq PE300 platform (Majorbio, Shanghai, China).

### Data processing and analysis

Following sequencing, the original sequences underwent quality control using Fastp (v0.19.6^1^) (Chen et al., 2018), with paired-end double-ended sequence splicing conducted using FLASH (v1.2.11^2^). The sequences were then clustered into operational taxonomic units (OTUs) at a 97% similarity threshold and chimeric sequences were removed using UPARSE (v7.1^3^) (Edgar, 2013). For species classification, the RDP classifier (v11.5^4^) was used against the SILVA database (Silva Release 138) with the threshold set to 0.8.

Rarefaction curves were constructed at the OTU level, which were generated to evaluate species abundance across samples with varying sequencing data, allowing for the assessment of the adequacy of sequencing data (Xu et al., 2014).

The relative abundance of bacterial taxa at the phylum and genus levels was quantified and presented as mean ± standard deviation (SD) using R tools. Differences in the top 15 dominant bacteria at the phylum and genus levels in the gut microbiota among the five temperature groups were analyzed using the Kruskal-Wallis rank-sum test, with false discovery rate (FDR) correction for *p*-values. Alpha diversity, reflecting community diversity (Shannon and Simpson indices) and community richness (Ace and Chao indices), was calculated using Mothur (v1.30.2^5^). The Kruskal-Wallis rank-sum test was used to evaluate differences in alpha diversity among the groups. The beta diversity distance matrix was computed using QIIME (v1.9.1^6^). Principal coordinate analysis (PCoA) based on unweighted and weighted UniFrac distance metrics was performed at the OTU level. Partial least squares discriminant analysis (PLS-DA) was performed to differentiate the gut microbiota among the different temperature groups. The differential abundances of microbiota at various taxonomic levels were compared and the effect size of each selected classification was evaluated using linear discriminant analysis (LDA) effect size (LEfSe) (Segata et al., 2011), with significance determined at LDA score > 4 and *p* < 0.05. Functional profiles of the gut microbiota were predicted using PICRUSt2 [phylogenetic investigation of communities by reconstruction of unobserved states 2 (v2.2.0^7^)] (Langille et al., 2013), and differences in functional Kyoto Encyclopedia of Genes and Genomes (KEGG) pathways among the different groups were determined using the Kruskal-Wallis rank-sum test with IBM SPSS (v23.0).

## Results

A total of 1,459,544 original sequences were obtained from the hypervariable V3–V4 region of the 16S rRNA gene across 35 samples. After undergoing quality control, the sequencing results were standardized based on the minimum number of sample sequences, then annotated to obtain a total of 23 phyla, 48 classes, 121 orders, 222 families, 478 genera, 680 species, and 1,060 OTUs. Analysis of the rarefaction curves indicated that the rarefaction curve tended to be flat and remained constant as sequencing data volume increased, suggesting that the amount of sequencing data was sufficient, reliable, and met the analytical requirements in terms of depth and accuracy (Supplementary Figure S1).

### General analyses of gut microbial community structure in crocodile lizards

**Figure 1:**
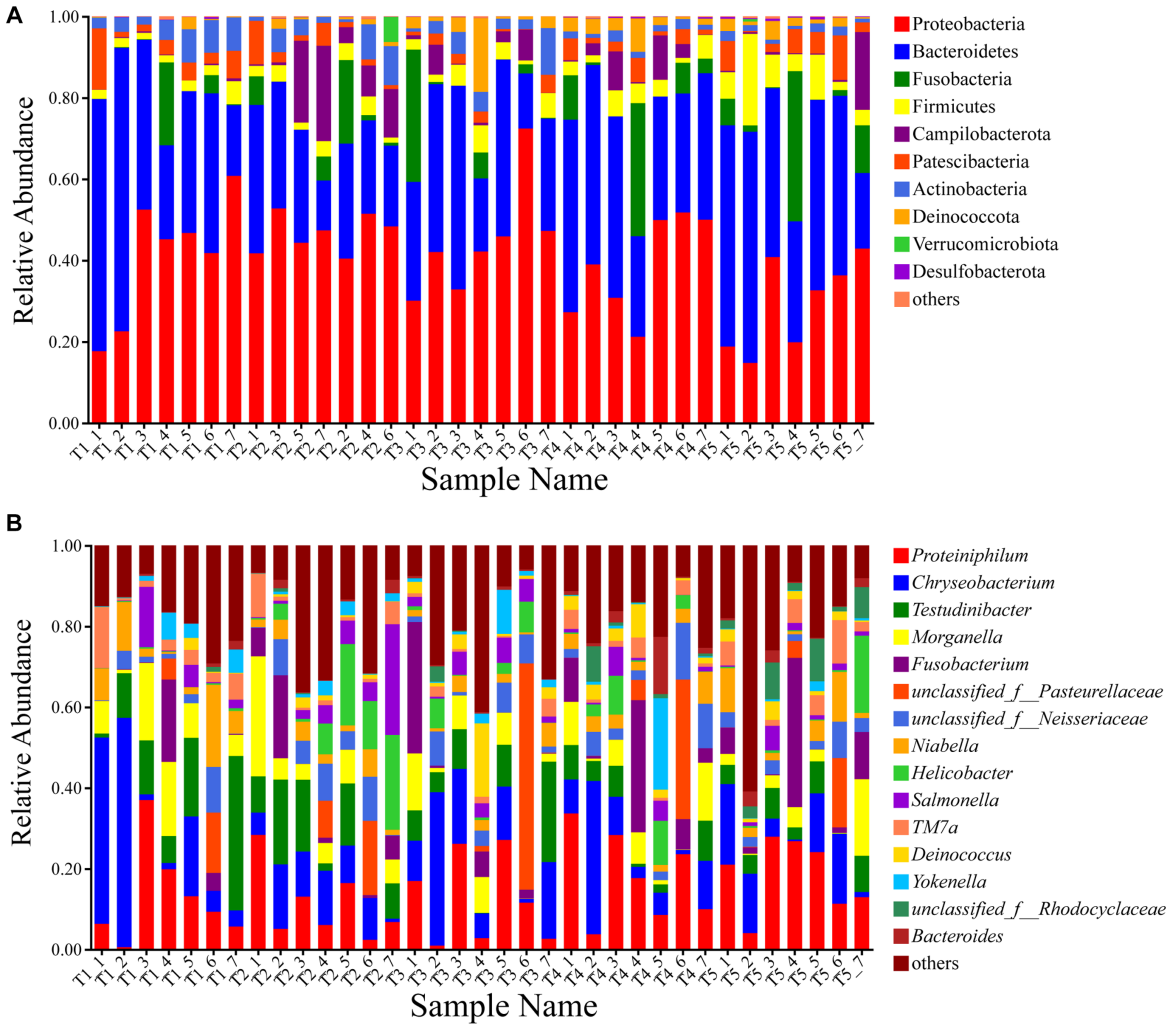
Composition of the gut microbiota of crocodile lizards at the phylum **(A)** and genus levels **(B)**.

Among the total sequences, most bacteria were classified into 10 phyla (Figure 1A). The dominant phyla in the gut microbiota of the experimental crocodile lizards included Proteobacteria (40.06% ± 6.72%) and Bacteroidetes (35.54% ± 6.83%), followed by Fusobacteria (6.19% ± 1.96%), Firmicutes (4.39% ± 2.25%), Campilobacterota (3.97% ± 3.50%), Patescibacteria (3.67% ± 1.29%), Actinobacteria (3.48% ± 1.72%), and Deinococcota (2.16% ± 1.57%) (Supplementary Table S4). The top 15 dominant genera in the gut microbiota of the experimental crocodile lizards were listed and the most abundant taxa were *Proteiniphilum* (14.62% ± 3.27%), *Chryseobacterium* (13.06% ± 4.08%), *Testudinibacter* (8.34% ± 3.41%), *Morganella* (6.72% ± 1.67%), and *Fusobacterium* (6.19% ± 1.96%) (Figure 1B; Supplementary Table S5).

### Differences in composition and abundance of gut microbiota

**Figure 2:**
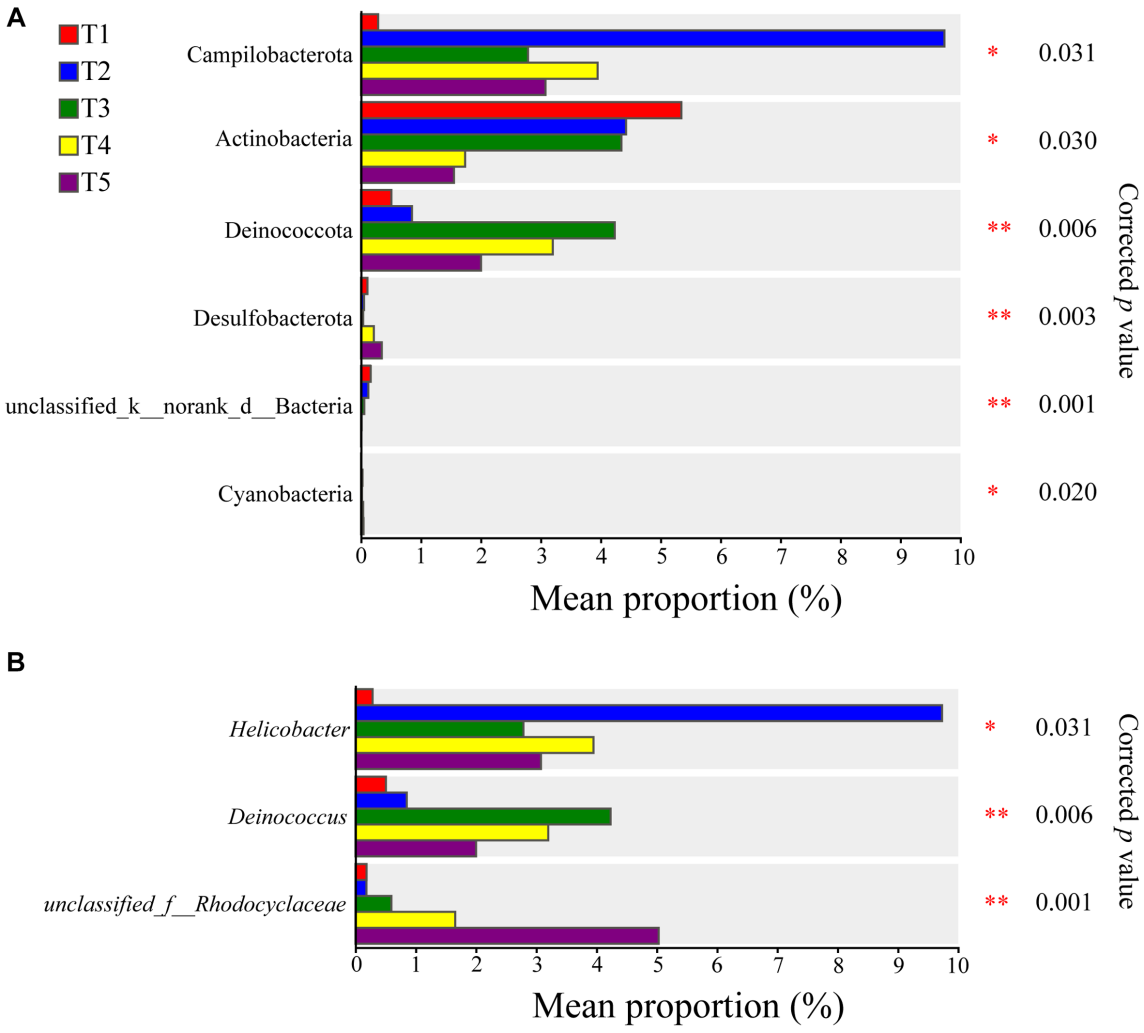
Differences of gut microbiota composition in crocodile lizards at the phylum **(A)** and genus levels **(B)**. *p ≤ 0.05, **p ≤ 0.01.

Based on the Kruskal-Wallis rank-sum test, significant differences were observed in the abundances of Campilobacterota, Actinobacteria, Deinococcota, Desulfobacterota, Unclassified_k__norank_d__Bacteria, and Cyanobacteria in the five temperature groups at the phylum level. Actinobacteria showed lower enrichment at higher temperatures, with a significantly higher relative abundance at 22°C compared to 28°C and 30°C (Supplementary Figure S2). At the genus level, *Helicobacter, Deinococcus*, and *unclassfied_f__Rhodocyclaceae* exhibited significant differences between the groups (Figure 2). The opportunistic pathogen *Pseudomonas aeruginosa* was also detected in the gut microbiota of the crocodile lizards. Based on the Kruskal-Wallis rank-sum test, significant differences were observed among the five temperature groups, with the relative abundance of *P. aeruginosa* found to be significantly higher at 30°C than at 22°C and 24°C (Supplementary Figure S3).

**Figure 3:**
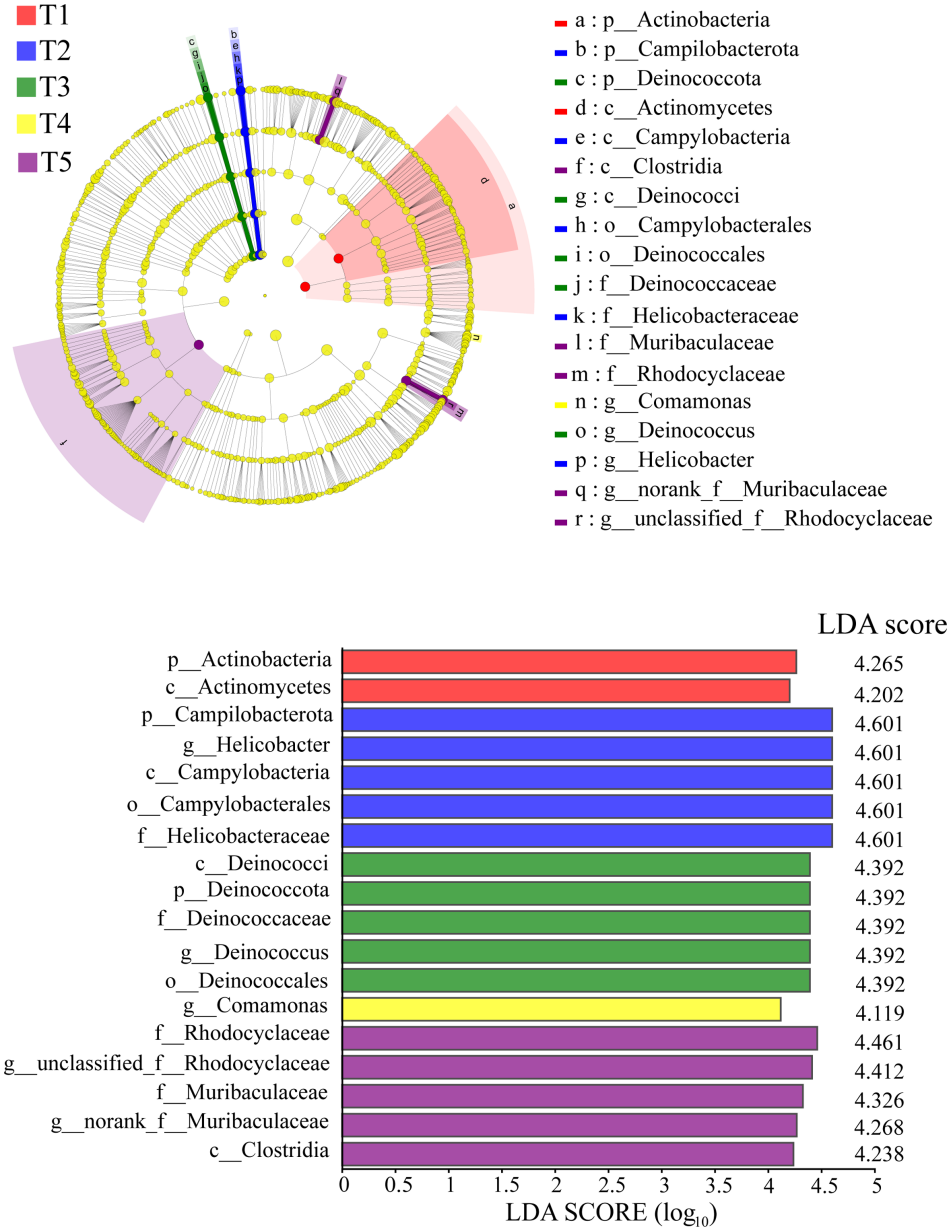
LEfSe analysis of the gut microbiota in crocodile lizards.

The LEfSe analysis identified significant differences in 18 intestinal bacterial taxa among the different groups (Figure 3). The LEfSe distribution bar chart displayed a positive correlation between the length of the bars and the significance of the differences observed in the taxa, with longer bars representing higher levels of significance.

### Differences in alpha and beta diversities of gut microbiota

**Figure 4:**
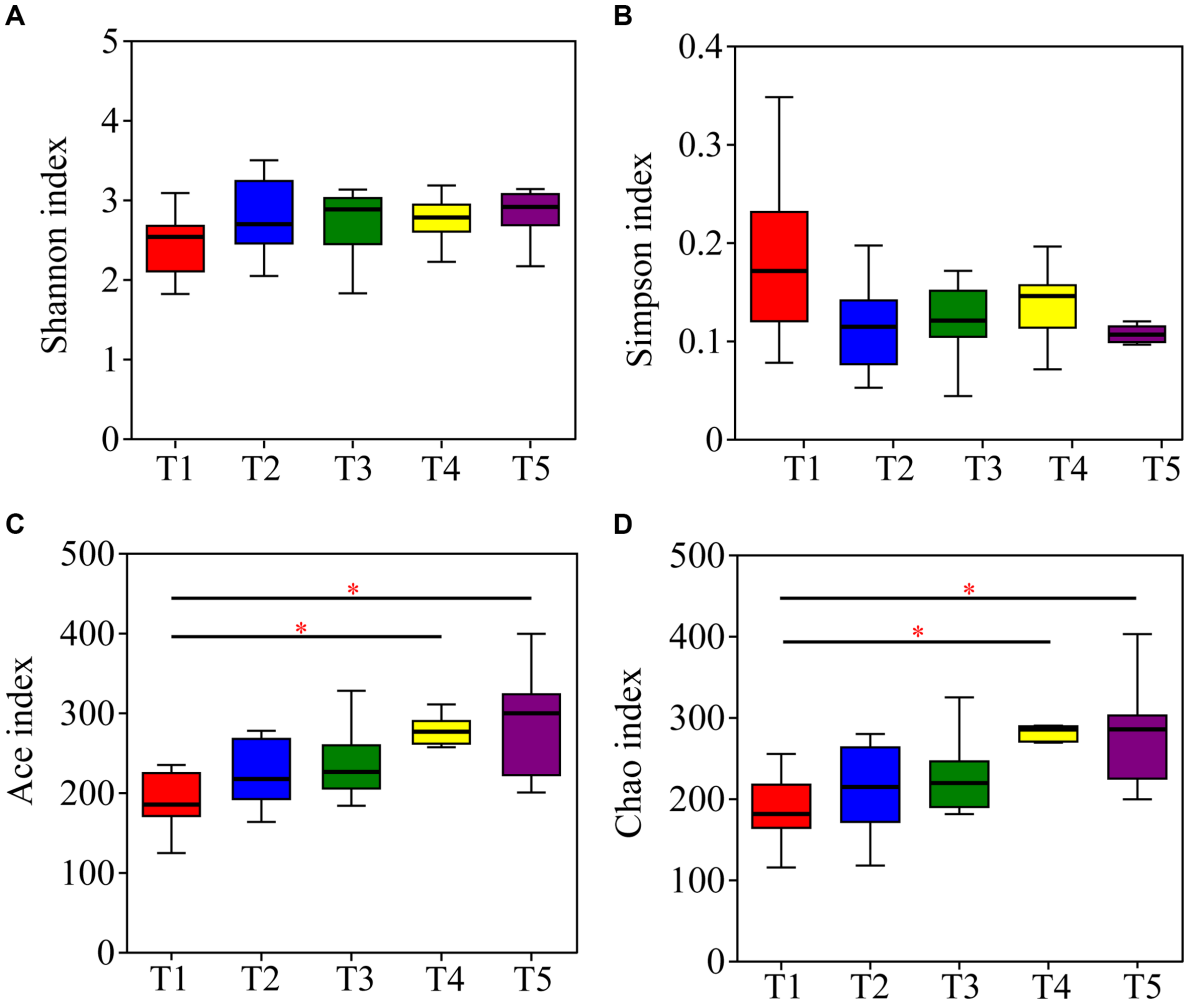
Differences in alpha diversity of gut microbiota in crocodile lizards at different temperatures. **(A)** Shannon index; **(B)** Simpson index; **(C)** Ace index; **(D)** Chao index. *p ≤ 0.05.

The Kruskal-Wallis rank-sum test showed no significant differences in community diversity in the different temperature groups (*p* > 0.05). However, significant differences in community richness were detected (*p* < 0.05), with significantly higher richness in the T4 and T5 temperature groups than in the T1 temperature group after the *post-hoc* test (Table 1; Figure 4).

**Table 1:** Alpha diversity of gut microbiota in crocodile lizards in different temperature groups.

Temperature group	T1 22°C	T2 24°C	T3 26°C	T4 28°C	T5 30°C	*p* value
Shannon	2.43 ± 0.44	2.80 ± 0.55	2.79 ± 0.66	2.75 ± 0.31	2.93 ± 0.55	0.455
Simpson	0.19 ± 0.10	0.11 ± 0.05	0.14 ± 0.09	0.14 ± 0.04	0.11 ± 0.05	0.338
Ace	190.46 ± 39.95	225.08 ± 46.80	237.89 ± 50.35	271.92 ± 31.19	283.96 ± 72.22	0.040
Chao	187.55 ± 46.54	211.27 ± 60.67	227.81 ± 50.81	283.34 ± 41.58	277.11 ± 69.66	0.007

**Figure 5:**
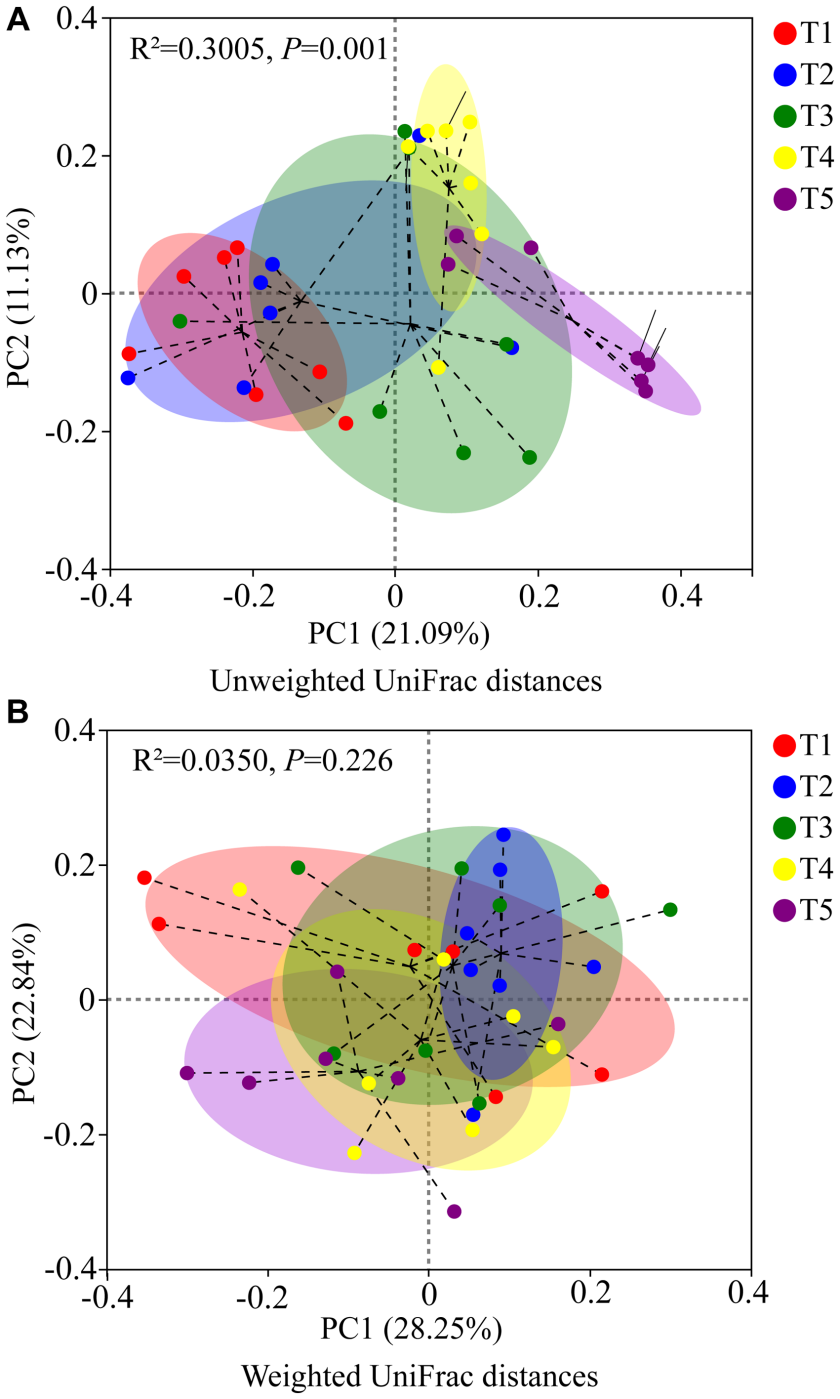
PCoA based on Unweighted UniFrac metrics **(A)** and Weighted UniFrac **(B)** of gut microbiota in crocodile lizards in different temperature groups.

**Figure 6:**
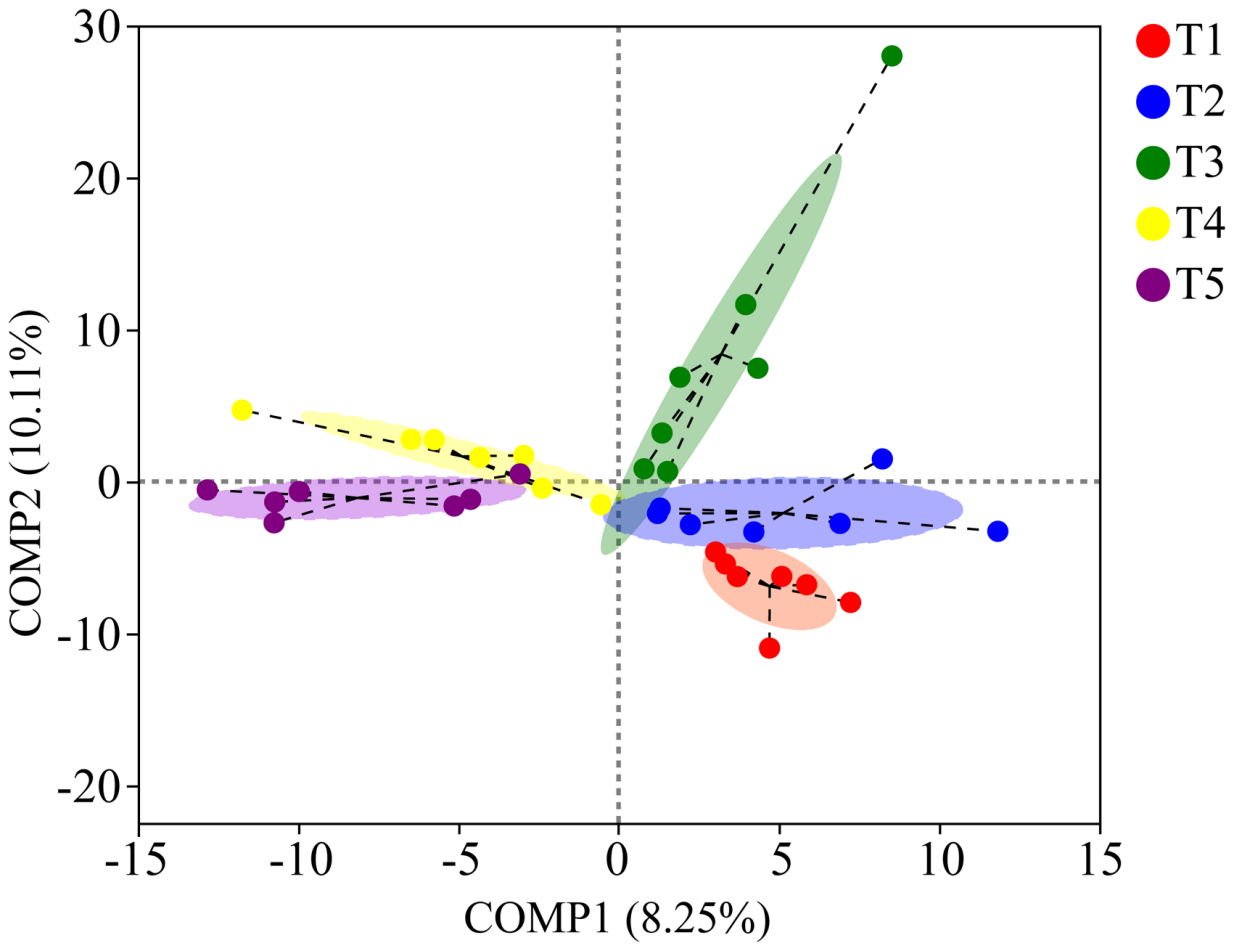
PLS-DA of gut microbiota in crocodile lizards at different temperature groups.

The PCoA results based on unweighted UniFrac metrics revealed distinct separation among the gut microbiota of crocodile lizards from the different temperature groups, but no observable separation between groups based on weighted UniFrac (Unweighted UniFrac: *R*^2^ = 0.3005, *p* = 0.001; Weighted UniFrac: *R*^2^ = 0.0350, *p* = 0.226) (Figure 5). The PLS-DA results demonstrated clear differentiation and clustering of the gut microbiota from crocodile lizards in the different temperature groups, indicating distinct structural variations among the gut microbiota at these temperatures (Figure 6).

### Functional profile prediction of gut microbiota

**Figure 7:**
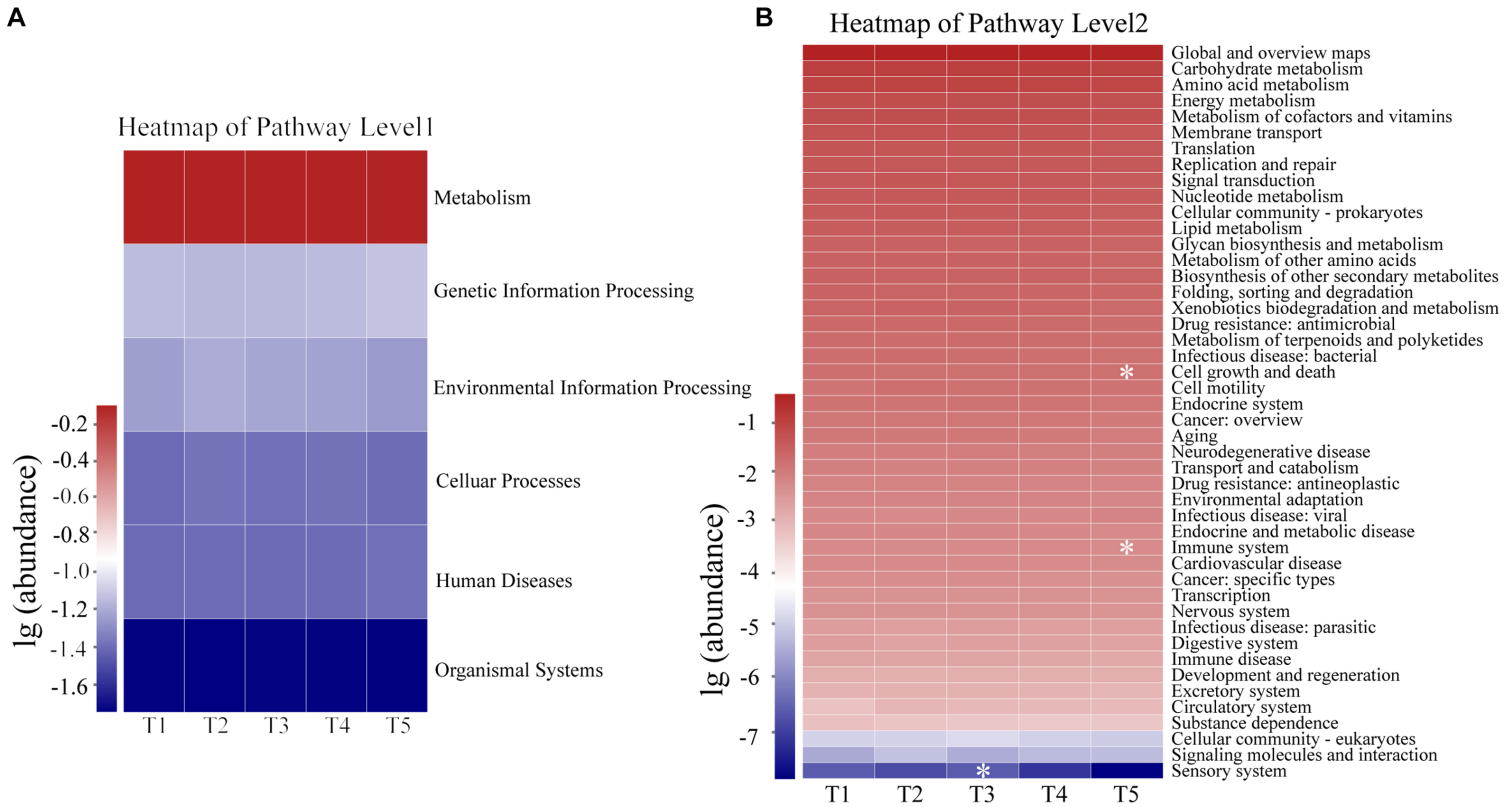
Functional profiles of Level 1 **(A)** and Level 2 **(B)** KEGG pathways of gut microbiota in crocodile lizards. *p ≤ 0.05.

According to the PICRUSt2 results, the primary functions of the gut microbiota were related to metabolism (79.72% ± 0.82%), followed by genetic information processing (7.28% ± 0.56%), environmental information processing (6.01% ± 0.77%), cellular processes (4.11% ± 0.42%), human diseases (4.04% ± 0.30%), and organismal systems (1.86% ± 0.10%) at the KEGG pathway level 1 (Supplementary Table S6). Furthermore, the Kruskal-Wallis rank-sum test revealed no significant differences in the six functional pathways of the gut microbiota among the five temperature groups. However, among the 46 functional pathways detected at KEGG pathway level 2, significant differences in the cell growth and death, immune system, and sensory system pathways were observed in the gut microbiota of the five temperature groups (Figure 7).

## Discussion

Previous studies on reptiles have consistently reported the predominance of Bacteroidetes, Firmicutes, and Proteobacteria in the gut microbiota of snakes (Costello et al., 2010), lizards (Zhang et al., 2018; Zhang Z. et al., 2022), and tortoises (Yuan et al., 2015). In crocodiles, however, the dominant phyla have been identified as Fusobacteria, Firmicutes, and Bacteroidetes (Keenan et al., 2013; Lin et al., 2019). In our study on crocodile lizards, we found Proteobacteria and Bacteroidetes to be the predominant phyla, in accordance with previous research exploring the relationship between the gut microbiota of crocodile lizards and diet and disease (Jiang et al., 2017). However, another study reported Firmicutes and Proteobacteria as the main phyla in crocodile lizards (Tang et al., 2020), with these variations potentially due to differences in sampling methods. Moreover, studies on the response of gut microbiota in *E. argus* to environmental temperatures reported relative stability of the main phyla within the gut microbiota following warming experiments (Zhang Z. et al., 2022). Similarly, research conducted on *Andrias davidianus* demonstrated that after acclimation to increased temperatures (Day 80), the two phyla with the highest relative abundance in the gut microbiota were Firmicutes and Fusobacteria (Zhu et al., 2021). Proteobacteria contribute to degrade a variety of aromatic compounds and boost the nutrient absorption of their host (Rowland et al., 2018). Bacteroidetes play an important role in degrading carbohydrates and proteins in the human large intestine (Nuriel-Ohayon et al., 2016; Rinninella et al., 2019). In our study, the dominant phyla in the gut microbiota of the crocodile lizards remained consistent in the different temperature groups, suggesting that the responses of the dominant microbiota to temperature may be conserved within the temperature range of 22°C–30°C, and their functions may be retained.

Temperature has been identified as a crucial factor influencing the composition of gut microbiota in amphibians and reptiles in various studies (Kohl and Yahn, 2016; Bestion et al., 2017; Fontaine et al., 2018; Li et al., 2020; Moeller et al., 2020). Consistent with these findings, our study revealed significant differences in the beta diversity and structure of the gut microbiota among the five temperature groups, indicating temperature-dependent changes in the community structure of gut microbiota in crocodile lizards. Interestingly, previous research on fish reported a greater richness of microbiome composition under warmer temperatures, potentially attributed to selective pressure (Kokou et al., 2018). Furthermore, an increase in microbial diversity and richness with temperature increases has been reported in *Rana chensinensis* tadpoles, possibly due to a higher prevalence of pathogenic taxa (Niu et al., 2022). In our study, we observed significantly higher community richness of the gut microbiota in crocodile lizards at 28°C and 30°C than at 22°C. This suggests that warmer temperatures may create an optimal and permissive environment for the growth and reproduction of gut microbiota in crocodile lizards, leading to greater microbial variability and richness. Our findings provide further evidence supporting the important role of temperature as a key factor influencing microbial community structure and richness.

Given the primary role of gut microbiota in host metabolism (Nicholson et al., 2012; Sepulveda and Moeller, 2020), our study also found that the dominant functional pathways of the gut microbiota in crocodile lizards was related to metabolism, paralleling findings in other studies on lizards (Tang et al., 2020; Du et al., 2022). Previous studies have also shown that temperature-induced changes in the gut microbiota can impact host metabolism and facilitate adaptation to thermal environments (Chevalier et al., 2015; Bestion et al., 2017; Worthmann et al., 2017; Chen et al., 2022). Firmicutes and Bacteroidetes, which are implicated in protein and nutrient metabolism as well as carbohydrate degradation, play a positive role in regulating host metabolism (Bernini et al., 2016; Colston and Jackson, 2016). Although an increased relative abundance of Firmicutes has been associated with heightened host metabolism and food consumption (Zhu et al., 2021), our study did not observe any significant differences in the abundance of Firmicutes and Bacteroidetes with rising temperature. Additionally, both food intake and the abundance of metabolic pathways remained relatively stable across the different temperature experiments. These results suggest that the gut microbiota related to metabolism may be able to regulate and acclimate to warming within the experimental temperature range, thereby maintaining metabolic function in the crocodile lizards. Furthermore, the experiment design, which consisted of a gradual shift across five temperature gradients, may also have contributed to the maintenance of gut microbiota stability and resilience in the crocodile lizards.

In addition to its role in host metabolism, the gut microbiota also plays an important role in providing protection against pathogen infection, with individuals showing low immune function often harboring an abnormal gut microbiota (Lozupone et al., 2013; Sepulveda and Moeller, 2020; Zhu et al., 2021; Siddiqui et al., 2022). Increasing ambient temperature is associated with a significant decrease in the relative abundance of microbiota that provide host protection through the production of antifungal metabolites, potentially compromising the ability of the host to resist pathogens (Becker et al., 2009; Fontaine et al., 2018). Our study revealed a significant decrease in the relative abundance of Actinobacteria with increasing temperature. These bacteria are known to produce a variety of antimicrobial secondary metabolites and serve as a major source of new antibiotics for pharmaceutical applications (Berdy, 2012; Jakubiec-Krzesniak et al., 2018). The positive effects of Actinobacteria-produced antibiotic compounds on host growth may disappear under increasing ambient temperature (Horvathova et al., 2019). Consequently, increasing temperatures may have a negative impact on the antibacterial capacity and gut homeostasis of crocodile lizards. To date, studies on the protective roles of Actinobacteria against animal pathogens have been limited to insect species (Kaltenpoth, 2009; Kaltenpoth and Engl, 2014). Thus, further investigations are necessary to comprehensively understand the regulatory effects of Actinobacteria in crocodile lizards. In contrast, *P. aeruginosa* is an opportunistic pathogen known to cause skin infections in humans and reptiles, including crocodile lizards (Milivojevic et al., 2018; Xiong et al., 2022). Here, we observed a higher relative abundance of *P. aeruginosa* at 30°C compared to 22°C and 24°C, indicating that elevated temperature may be beneficial for the survival of these bacteria. Thus, these findings suggest that an increase in temperature may negatively affect host health by affecting the abundances of Actinobacteria and *P. aeruginosa*. Therefore, it is important to implement appropriate cooling measures and strengthen disease prevention during the high-temperature season to reduce the risk of infections in crocodile lizards. Furthermore, considering that these pathogens can spillover to humans, it is recommended that personal hygiene be maintained before and after contact with crocodile lizards.

## Conclusion

In conclusion, our study revealed that temperature has a significant impact on the microbial community structure of crocodile lizards. Notably, our results showed that community richness increased significantly with increasing temperatures, while community diversity and dominant microbiota were largely retained. Furthermore, metabolic function and related microbiota also remained relatively stable across the different temperature groups, possibly due to regulatory and acclimation processes. However, notable changes in the abundances of *P. aeruginosa* and Actinobacteria in response to warming may have implications for the health and physiology of crocodile lizards. These findings provide valuable insights into the responses and ecological adaptations of crocodile lizards to warming. In future research, it will be important to identify the potential mechanisms underlying of response of microbiota to warming and further understand the contributions of microbial alterations to the hosts in their natural environment.

## Data availability statement

The datasets presented in this study can be found in online repositories. The names of the repository/repositories and accession number(s) can be found at: https://www.ncbi.nlm.nih.gov/, PRJNA993385.

## Ethics statement

The animal study was reviewed and approved by Laboratory Animal Care and Animal Ethics Committee of Guangxi Normal University. The studies were conducted in accordance with the local legislation and institutional requirements.

## Author contributions

ZW: Funding acquisition, Methodology, Project administration, Resources, Writing – review & editing. MH: Conceptualization, Data curation, Formal analysis, Investigation, Writing – original draft. ZL: Conceptualization, Data curation, Formal analysis, Investigation, Writing – original draft. CZ: Investigation, Methodology, Writing – original draft. YL: Writing – review & editing. ST: Data curation, Writing – original draft. XK: Formal analysis, Investigation, Writing – original draft.

## Footnotes

^1^ https://github.com/OpenGene/fastp

^2^ https://ccb.jhu.edu/software/FLASH/index.shtml

^3^ http://www.drive5.com/uparse/

^4^ https://sourceforge.net/projects/rdp-classifier/

^5^ https://www.mothur.org/wiki/Download_mothur

^6^ http://qiime.org/install/index.html

^7^ https://github.com/picrust/picrust2/
