# Supplementary material for: Responses of gut microbiota in crocodile lizards (Shinisaurus crocodilurus) to changes in temperature
Source: Front Microbiol. 2023 Nov 15;14:1263917. doi: 10.3389/fmicb.2023.1263917 (PMC10684959; doi:10.3389/fmicb.2023.1263917)
Supplement: Supplementary file 7 [file Data_Sheet_1.DOCX]

***Supplementary Material***

**Responses of Gut Microbiota in Crocodile Lizards (*Shinisaurus crocodilurus*) to Changes in Temperature**

**Zhengzhong Lin^1, 2†^, Mingxian He^3†^, Chunying Zhong^4^, Yuhui Li^1, 2^, Sanqi Tang^1, 2^, Xindan Kang^1, 2^, Zhengjun Wu^1, 2^***

*** Correspondence:** Corresponding Author: wu_zhengjun@aliyun.com

1. **Supplementary Figures and Tables**
   1. **Supplementary Tables**

Supplementary Table 1. Morphological indicators and birth dates of crocodile lizards.

Supplementary Table 2. food intake of crocodile lizards in 5 temperature groups.

Supplementary Table 3. Body temperature of crocodile lizards at 5 temperature groups

Supplementary Table 4. The differences in the relative abundance of gut microbiota in 5 temperature groups (at phylum level).

Supplementary Table 5. The differences in the relative abundance of gut microbiota in 5 temperature groups (top 50 dominant bacteria at genus level).

Supplementary Table 6. The functional profiles of pathways Level 1 of gut microbiota in crocodile lizards in 5 temperature groups.

- 1. **Supplementary Figures**


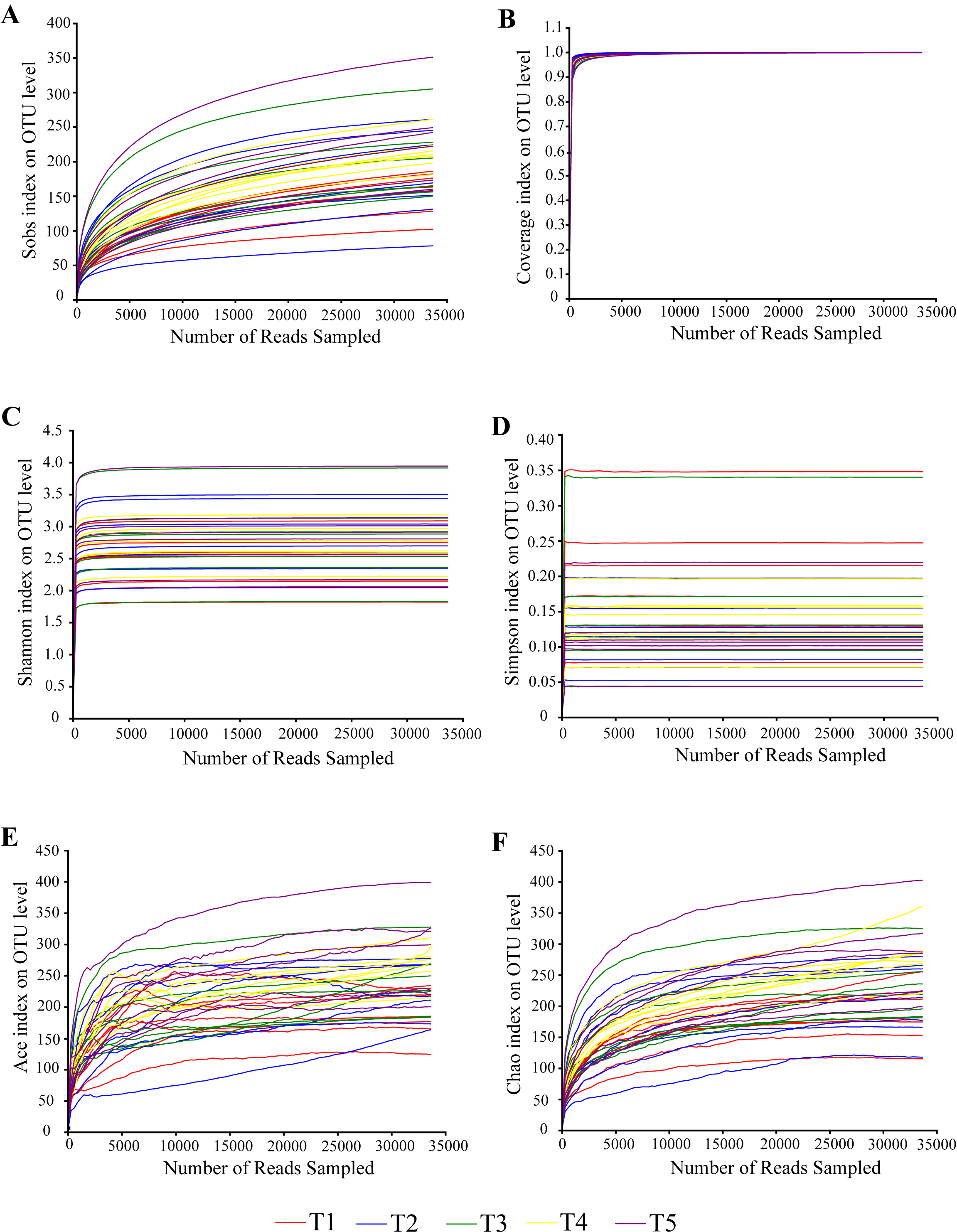
Supplementary Figure 1. Rarefaction curves and rank-abundance curves


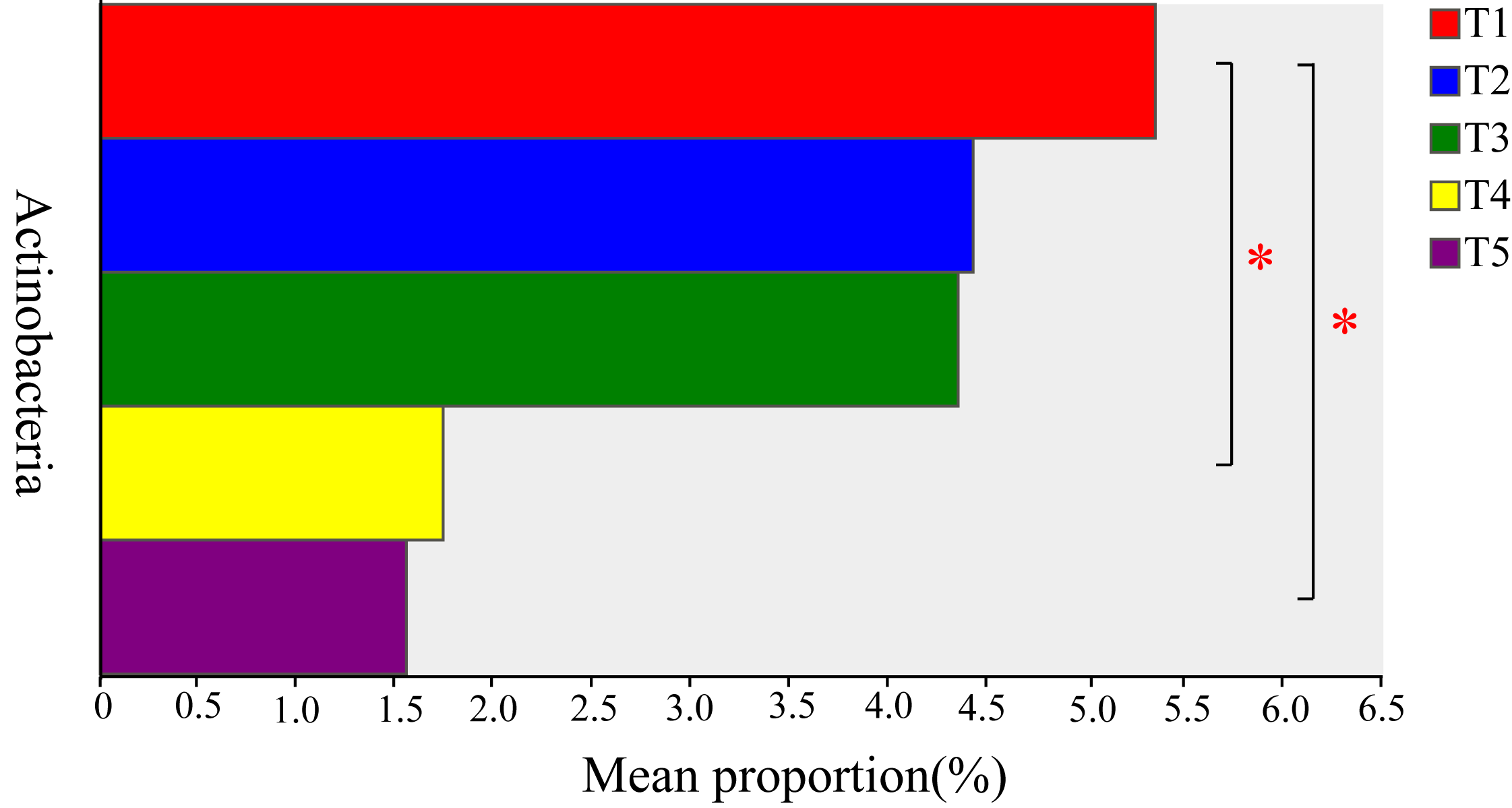
Supplementary Figure 2. Difference in the relative abundance of Actinobacteria at different temperatures


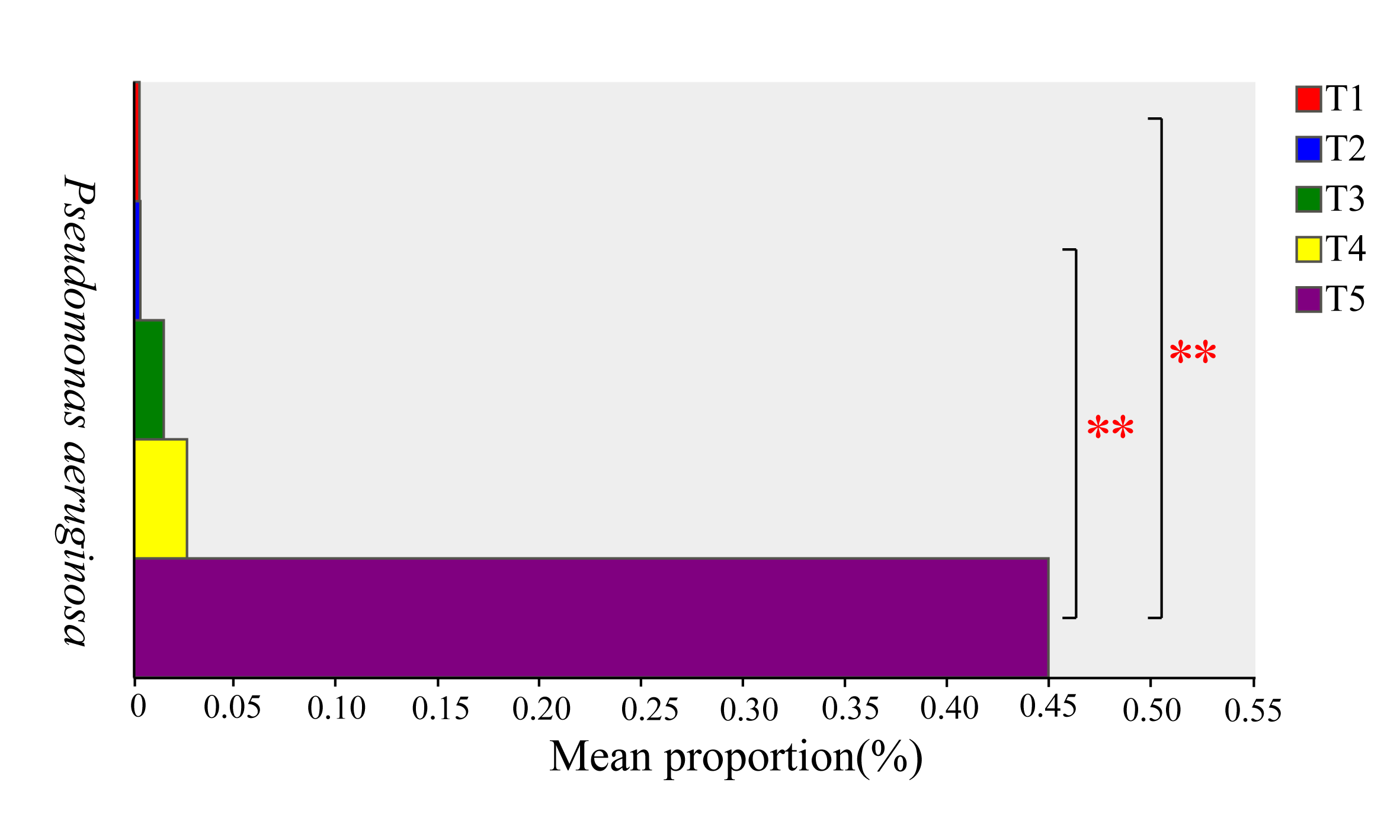
Supplementary Figure 3. Difference in the relative abundance of *Pseudomonas aeruginosa* at different temperatures
