# Supplementary figures and images for: Responses of gut microbiota in crocodile lizards (Shinisaurus crocodilurus) to changes in temperature
Source: Front Microbiol. 2023 Nov 15;14:1263917. doi: 10.3389/fmicb.2023.1263917 (PMC10684959; doi:10.3389/fmicb.2023.1263917)

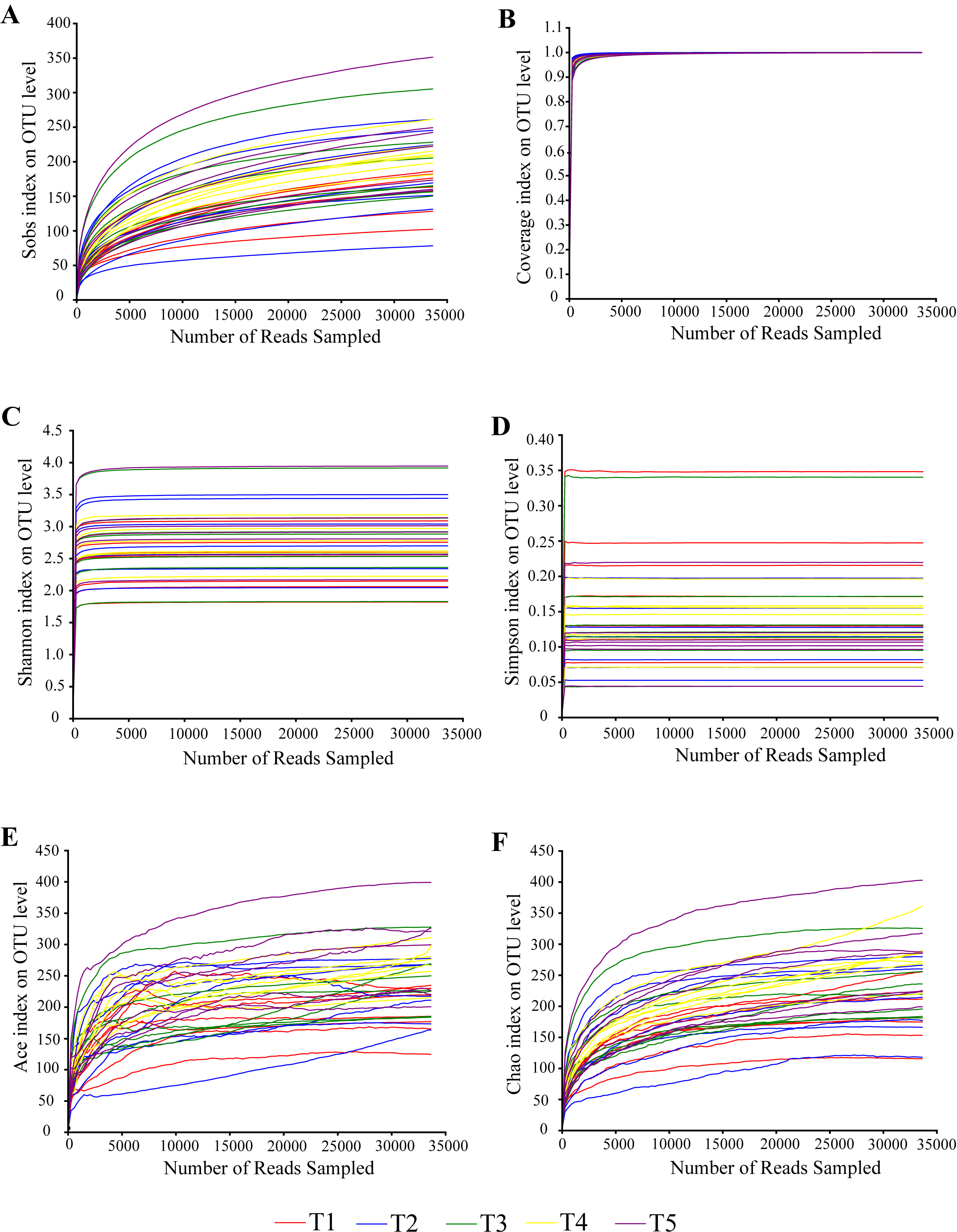

Supplement: Supplementary file 8 [file Image_1.tif]

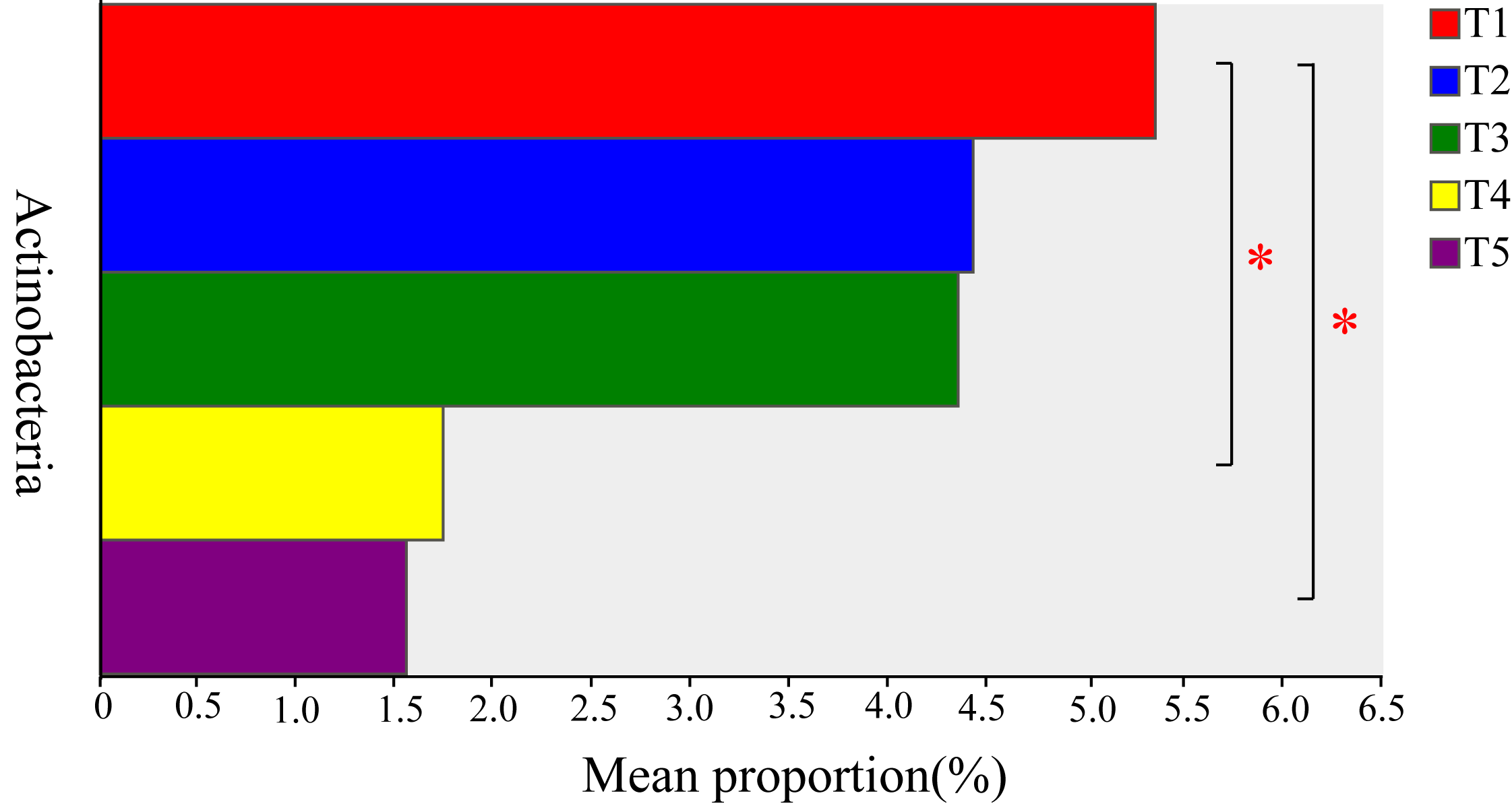

Supplement: Supplementary file 9 [file Image_2.tif]

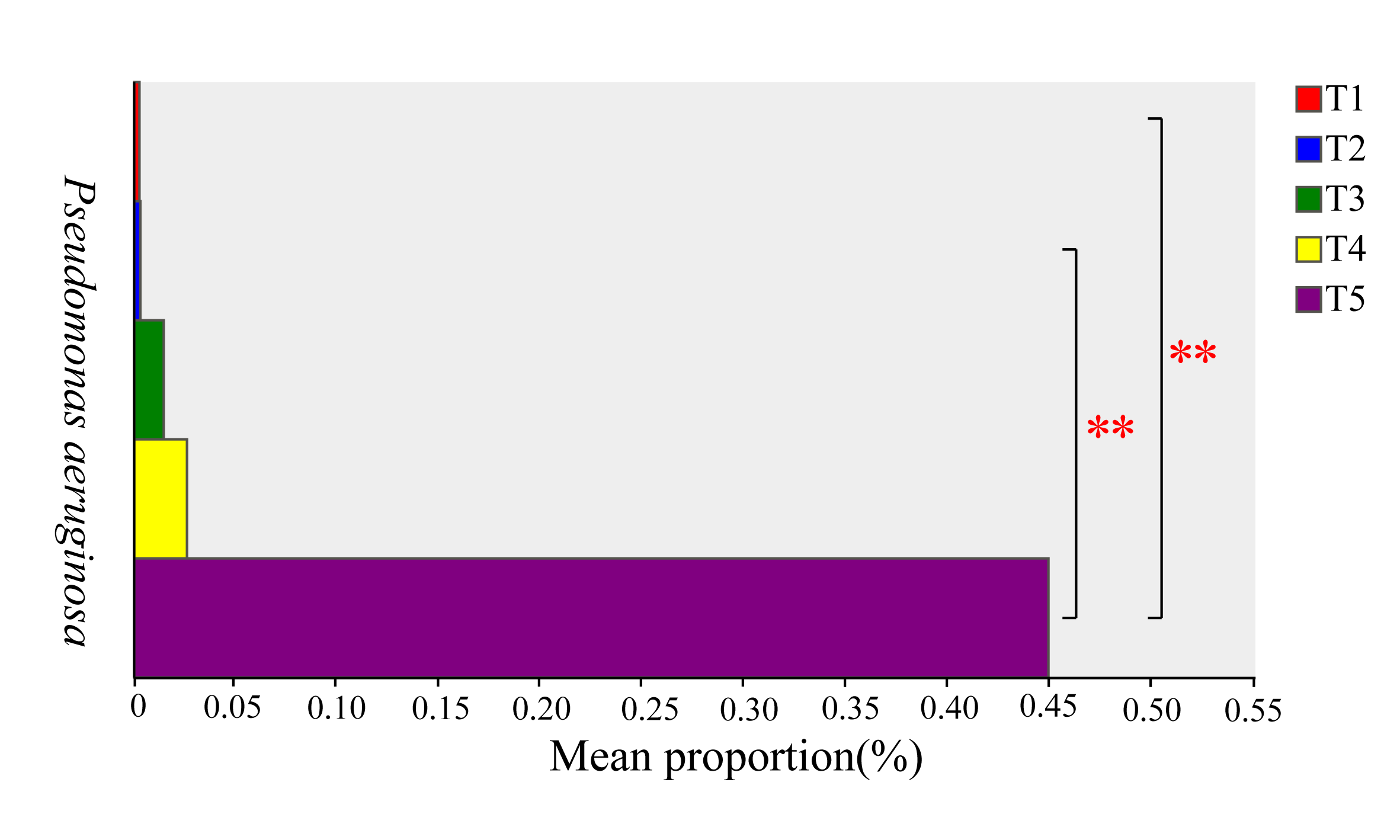

Supplement: Supplementary file 10 [file Image_3.tif]
